# Are Physical Education Policies Working? A Snapshot From San Francisco, 2011

**DOI:** 10.5888/pcd10.130108

**Published:** 2013-08-22

**Authors:** Hannah R. Thompson, Jennifer Linchey, Kristine A. Madsen

**Affiliations:** Author Affiliations: Hannah Thompson, University of California, San Francisco, Department of Pediatrics; Jennifer Linchey, University of California, Berkeley, School of Public Health, Berkeley, California. Dr Madsen is also affiliated with the University of California, San Francisco, Department of Pediatrics.

## Abstract

**Introduction:**

School physical education (PE) has been identified as a critical public health tool to increase physical activity among youths. We sought to objectively assess compliance with PE quantity mandates and quality recommendations in a large urban California school district.

**Methods:**

We collected PE schedules and systematically observed PE lessons (n = 154) in 20 elementary, 4 middle, and 4 high schools from February through May 2011.

**Results:**

On the basis of schools’ master schedules, 83% of elementary schools met the California state mandate of 100 PE minutes per week. Teachers' actual schedules indicated that 20% of schools met the mandate, and observation showed that only 5% were in compliance. All middle and high schools met the mandated 200 minutes per week. On average, classes at all school levels met the recommended 50% of PE lesson time in moderate-to-vigorous physical activity. No teacher- or school-level factors significantly predicted PE quantity, but credentialed elementary PE teachers spent more time building students’ motor skills.

**Conclusions:**

Our results suggest that current national estimates of PE, which are based on schools’ self-report, overestimate the amount of PE provided in elementary schools. Although more than half of PE class time was spent in moderate-to-vigorous physical activity, total physical activity in elementary schools from PE is minimal and may do little to contribute to students’ overall health.

## Introduction

Despite the many health benefits of physical activity (1), few youths achieve recommended levels of moderate-to-vigorous physical activity (2). Increasing physical education (PE) in school is an optimal strategy for increasing physical activity for students (3). In addition to providing structured moderate-to-vigorous physical activity, PE supports acquisition of skills, knowledge, and behaviors that may facilitate a lifetime of physical activity (4).

California state policy mandates an average of 100 minutes of PE every week for elementary school students and 200 minutes per week for middle and high school students (5). Yet many schools do not comply with this mandate, particularly elementary schools (6). Additionally, data on PE frequency are based on administrators’ reports, not on objective measures (6,7). Thus, the actual degree of compliance with policy is unknown.

The US Department of Health and Human Services recommends that students spend 50% of PE time in moderate-to-vigorous physical activity (8). Credentialed PE teachers (PE specialists) have been shown to deliver greater levels of moderate-to-vigorous physical activity than classroom teachers (9). However, because of budget cuts and low prioritization of PE, many schools rely on classroom teachers to teach PE (10). In some cases, part-time PE specialists teach PE and also build classroom teachers’ capacity to lead PE through training because many classroom teachers have no formal training in PE. Little evidence exists comparing PE delivered by specialists versus PE delivered by trained and untrained classroom teachers, yet these configurations likely influence the quality and quantity of PE students receive.

The objective of this study was to determine if schools in a racially and ethnically diverse urban school district met California’s PE requirements and to assess differences in PE quantity, physical activity level, and lesson content in PE led by specialists versus trained and untrained teachers.

## Methods

This mixed methods observational study took place from February through May 2011 in the San Francisco Unified School District (SFUSD), an urban district with nearly 56,000 students; 75% of these students were of African American, Latino, and Asian race/ethnicity and 60% qualified for free or reduced-price meals (11). The SFUSD research department and the UCSF Committee on Human Research approved all study procedures.

### Sample

The school district we studied comprised 72 elementary schools, 13 traditional middle schools, and 11 traditional high schools. Thirty-six of the elementary schools had access to a PE specialist, and all middle and high schools had at least one full-time PE specialist.

Because assessing differences between specialist and nonspecialist schools was a primary study aim, we selected 20 elementary schools for inclusion by using stratified random selection based on the presence of a PE specialist (10 with a specialist, 10 without). Sample size calculations (taking the design effect into account) were based on data from an unpublished study in a different school district (H.R.T. and K.A.M., unpublished data, 2010) and suggested that 10 schools per group would allow us to detect a 5-minute difference in moderate-to-vigorous physical activity with 80% power.

Differences in PE based on the presence of a specialist were not relevant in middle and high schools, all of which had specialists; therefore, a smaller sample of 4 middle and 4 high schools was selected on the basis of current students’ average aerobic capacity scores from the previous 3 school years (fitness testing is mandatory in California in grades 5, 7, and 9). We selected the 2 schools with the lowest scores, one with scores closest to the 50% percentile and one with the highest scores, to obtain a sample representing the range of student fitness performance across the district.

Principals at schools selected for inclusion were invited to participate in the study, and all principals assented. Study measures involved observations of PE classes for grades 5, 7, and 9 and interviews with all observed teachers.

Schools applied to the school district for a PE specialist and were selected on the basis of need and the school’s willingness to adopt the PE specialist program, which included a part-time PE specialist, district-adopted curriculum, equipment, and professional development. PE specialists provided lesson planning assistance, equipment and behavior management techniques, and strategies for implementing quality physical education. PE specialists worked full time and rotated among 1 to 3 schools, teaching at each school from 1 to 5 days per week and reaching each classroom of students an average of once per week. All PE specialists had a teaching credential with a specialty in PE and received approximately 70 hours of district-led PE professional development annually. In the 36 elementary schools without a district-provided PE specialist, PE was either taught by classroom teachers or by an adult with physical activity experience, such as coaching, but no teaching credential (hereafter called a PE leader).

Researchers observed a maximum of 2 adults (PE specialists or PE leaders) per elementary school, depending on the number of teachers teaching 5th grade PE (Figure). At the 10 schools with a PE specialist, researchers observed classes taught by the specialist and 1 randomly selected 5th grade classroom teacher. In the 10 schools without PE specialists, we observed a PE leader if one was present and also observed randomly selected classroom teachers.

**Figure:**
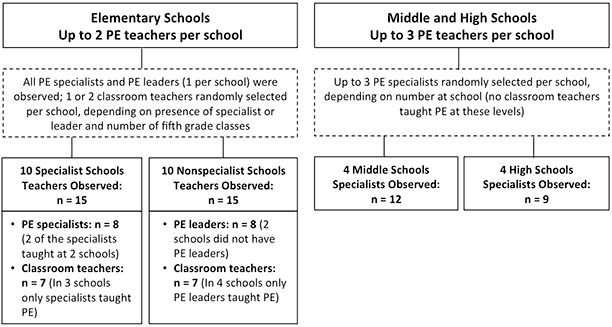
Selection of fifth-, seventh-, and ninth-grade physical education (PE) teachers for observations. A PE specialist is credentialed teacher with a specialty in physical education. A PE leader is an adult who has no teaching credentials but has experience in teaching physical activities, such as coaching.

Researchers observed up to 3 PE specialists each in both the 7th and 9th grades in the 4 middle and 4 high schools. When more than 3 PE specialists taught PE, we randomly selected specialists for observation (Figure).

### Measures

The school district’s PE department provided researchers with schedules for all PE specialists at the beginning of the school year. Researchers obtained school-level PE master schedules when available, which contain times for all PE classes in the school. Because elementary school teachers’ PE schedules often differed from the master schedule, we also contacted all fifth grade classroom teachers individually to obtain their personal PE schedules.

We used the System for Observing Fitness Instruction Time (SOFIT) (12) to collect data on observed lesson length, proportion of the lesson consisting of moderate-to-vigorous physical activity, and the lesson context in which activity occurred. Detailed SOFIT methodology has been described elsewhere (12). Prompted by an instructional audio recording, observers coded activity levels of 4 randomly selected students (2 girls and 2 boys) using momentary time sampling (alternating 10-second observe, 10-second record intervals) for the entire PE lesson. Activity levels (coded 1, lying; 2, sitting; 3, standing; 4, walking; and 5, very active) have been validated by using accelerometry (13). Physical activity levels 4 and 5 are considered moderate and vigorous, respectively. Lesson context (classroom management, knowledge acquisition, fitness, skill drills, game play, free play, and other activities) was recorded simultaneously with activity levels. The scheduled lesson length was noted and the observed lesson length was recorded (the number of minutes that PE actually occurred, with observations beginning when 50% of students had entered the PE area and stopping at the lesson’s termination, per SOFIT protocol).

### Procedures

For each teacher, researchers observed PE lessons on 3 randomly selected days on which PE was scheduled. If a lesson did not occur, that lesson was considered a “no-show,” and the researcher returned to observe on another random day. In elementary schools, 2 classroom teachers in schools without PE specialists and 7 classroom teachers in schools with PE nonspecialists had no preset PE schedule, so researchers set up specific times to observe PE lessons. These lessons were not included in the proportion of no-shows.

After PE observations were complete, we asked teachers questions adapted from the Physical Education module of the School Physical Activity Policy Assessment (14) regarding their training, years of experience with PE, and perceived support from their administration for PE.

### Data analysis

We used linear mixed models to examine the primary outcomes of scheduled lesson length, observed lesson length, and proportion of lesson time spent in moderate-to-vigorous physical activity (based on SOFIT data). We used logistic mixed models to examine the secondary outcome of time spent in specific lesson contexts. Given that PE specialists trained classroom teachers at specialist schools, we considered the presence of a specialist and teacher type within elementary schools as predictors in separate models. We also examined school type (elementary, middle, or high) as a predictor. We used linear mixed models to examine associations between teacher-level characteristics and PE quantity. All mixed models included random effects for teacher and school to account for correlations within these domains. All analyses were performed using Stata/MP version 11 (StataCorp LP, College Station, Texas).

## Results

School demographic data did not differ significantly between elementary schools with PE specialists and those with PE nonspecialists or by school level with the exception of student enrollment, which differed among elementary, middle, and high schools (Table 1).

**Table 1:** School Demographic Characteristics^a^, Evaluation of Effectiveness of Physical Activity Policies, San Francisco, California, 2010–2011 School Year

Demographic Characteristic	Elementary Schools				Middle Schools (n = 4)	High Schools (n = 4)
	All (n = 20)	Specialist Schools (n = 10)	Nonspecialist Schools (n = 10)	*P* Value^c^		
No. of students, mean (SD)	391 (150)	363 (150)	419 (153)	.42	739 (360)^d^^,^ e	1,232 (900)^d^
Students eligible for free or reduced price meals, % (SD)	60 (22)	59 (22)	61 (23)	.85	69 (14)	65 (17)
Race/ethnicity, % (SD)^b^						
African American	12 (14)	16 (11)	9 (16)	.27	11 (8)	13 (8)
Asian	30 (29)	30 (27)	29 (31)	.94	33 (32)	41 (17)
Latino	29 (25)	21 (17)	37 (29)	.17	37 (30)	24 (15)
White	13 (11)	14 (11)	13 (11)	.82	10 (6)	6 (6)
Academic Performance Index base score , mean (SD)	812 (92)	804 (100)	820 (88)	.70	753 (114)	715 (163)
Students in Healthy Fitness Zone for Aerobic Capacity^f^, % (SD)	63 (17)	62 (16)	64 (20)	.80	63 (19)	58 (18)

a Demographic information from the 2010–2011 school year.

b Race/ethnic groups with fewer than 5% of students (eg, American Indian, other, declined to state) not shown.

c *P* value for difference in means between specialist and nonspecialist elementary schools calculated by *t* test; *P* < .05 indicates significance.

d Indicates a significant difference in means between elementary and middle/high schools.

e Indicates a significant difference in means between middle and high schools.

f The state-wide fitness test, the FITNESSGRAM, uses Healthy Fitness Zones to evaluate fitness performance of fifth, seventh, and ninth graders. These zones are criterion-referenced standards and represent minimum levels of fitness for age and sex that offer protection against the diseases that result from sedentary living. Aerobic capacity reflects the maximum rate of oxygen uptake and use during exercise.

Researchers observed a total of 154 PE lessons: 91 fifth-grade lessons (27 specialist observations and 21 classroom teacher observations at specialist schools, and 43 nonspecialist observations at nonspecialist schools), 36 seventh-grade lessons, and 27 ninth-grade lessons.

### Elementary schools

On the basis of teachers’ PE schedules, only 4 (20%) of the elementary schools observed met the California state mandate of 100 minutes of PE per week, 3 of which were specialist schools. Master PE schedules were available at all 10 specialist and 2 nonspecialist schools. Although master PE schedules for the 10 specialist schools showed 100 scheduled minutes of PE per week, teachers’ individual schedules reflected 78 minutes per week. The 2 master schedules at the nonspecialist schools reflected an average of 88 minutes of PE per week while the teachers’ schedules reflected 84 minutes per week; the remaining 8 teachers’ schedules reflected 71 minutes of PE per week.

Overall, 33% of PE class observations resulted in no-shows, excluding lessons that were cancelled because of schedule conflicts, such as standardized or fitness testing or inclement weather (11 teachers had 1 no-show, 1 teacher had 2 no-shows, and 1 teacher had 3 no-shows). Recurrent reasons for no-shows included school events, field trips, and teacher absences. On the basis of observations, only 1 (a nonspecialist school) of 20 elementary schools met the mandated 100 minutes of PE per week.

When PE lessons did occur, students spent 54% of observed lesson time in moderate-to-vigorous physical activity, an average of 17 minutes per lesson. The proportion of observed lesson time in moderate-to-vigorous physical activity did not significantly differ between specialist and nonspecialist schools (Table 2) or across teacher type (Table 3). Average observed lesson length was 5 minutes shorter than scheduled lesson length (*P* < .001) (Table 2), resulting in an average of 70 minutes of actual exposure to PE per week. Accounting for both no-shows and actual lesson length, elementary students received an average of 45 minutes of PE per week.

**Table 2:** Physical Education Lesson Time in Moderate-to-Vigorous Physical Activity, Evaluation of Effectiveness of Physical Activity Policies, San Francisco, 2010-2011 School Year

Lesson Time	Elementary Schools^a^								Middle Schools (n = 4)		High Schools (n = 4)	

	All Schools (n = 20)		Specialist Schools (n = 10)		Nonspecialist Schools (n = 10)				Mean (SD)	Range	Mean (SD)	Range

	Mean (SD)	Range	Mean (SD)	Range	Mean (SD)	Range	*P* Value^b^	95% CI				
Scheduled lesson length (min)	36.4 (7.4)	25.0–55.0	35.6 (6.6)	25.0–55.0	37.3 (8.1)	30.0–55.0	.40	−7.2 to 2.9	50.1 (2.1)	40.0–57.0	58.0 (20.0)^c^	30.0–95.0
Observed lesson length (min)	31.4 (7.9)	10.0–53.7	32.0 (7.6)	10.0–50.0	30.7 (8.3)	12.3–53.7	.62	−3.6 to 6.1	39.9 (3.6)	28.0–48.3	48.7 (17.8) c>	20.0–90.3
Observed proportion of lesson time in MVPA, %	54.0 (12.9)	22.1–81.0	55.0 (12.9)	22.1–80.0	53.0 (12.9)	25.0 - 81	.55	−.02 to .12	51.2 (12.0)	26.5–76.0	55.7 (18.4)	0.7–79.1
Calculated minutes of MVPA per lesson, no.	16.6 (4.6)	6.0–26.3	17.2 (4.6)	7.0–26.3	15.9 (4.5)	6.0–24.7	.36	−1.3 to 3.6	20.4 (5.2) c ^, ^ d	10.0–31.3	27.5 (14.1) c	0.3–53.3

Abbreviation: SD, standard deviation; MVPA, moderate to vigorous physical activity.

a A maximum of two 5^th^ grade teachers were observed per elementary school, and a maximum of 3 7^th^ or 9^th^ grade teachers were observed per school for middle and high schools; all teachers were observed up to 3 times each.

b *P* value and 95% confidence interval for difference in means between specialist and non-specialist elementary calculated by linear mixed effects models accounting for clustering by teacher and school; *P* < .05 indicates significance.

c Significant difference in means between elementary and middle/high schools,

d Significant difference in means between middle and high schools.

e Lesson observation began when 50% of students had arrived at the physical education area.

**Table 3:** Characteristics of Physical Education Lessons Taught by Classroom Teachers^a^ in Elementary Schools, by School Type^b^, Evaluation of Effectiveness of Physical Activity Policies, San Francisco, California, 2010–2011 School Year

Lesson Characteristic	Specialist Schools (n = 10), Classroom Teachers (n = 7), mean (SD)	Range	Nonspecialist Schools (n = 10), Classroom Teachers (n = 7), mean (SD)	Range	*P* Value^b^
Scheduled lesson length (min)	36.4 (8.4)	30.0–55.0	37.1 (8.2)	30.0–55.0	.88
Observed lesson length(min)	30.7 (9.0)	14.7–50.0	29.1 (8.3)	12.3–47.3	.71
Observed^c^ proportion of lesson time in MVPA, %	55.4 (14.9)	24.3–79.5	51.6 (9.7)	36.5–73.1	.49
Calculated minutes of MVPA per lesson	16.5 (5.1)	8.3–26.0	14.9 (4.4)	7.0–22.0	.41
**Proportion of time spent in lesson contexts, % (SD)**					
Management	28.9 (45.3)	NA	23.4 (42.4)	NA	.06
Knowledge	12.8 (33.4)	NA	8.5 (27.9)	NA	.45
Fitness activity	15.3 (36.0)	NA	21.9 (41.3)	NA	.68
Skill drills	19.2 (39.4)	NA	2.5 (15.5)	NA	.03
Game play	17.8 (38.3)	NA	38.1(48.6)	NA	.05
Free play/other	6.0 (23.8)	NA	5.6 (23.0)	NA	.54

Abbreviation: MVPA, moderate to vigorous physical activity; NA, not applicable.

a Teachers were observed 3 times each for a total of 21 observations at specialist schools and 21 observations at nonspecialist schools.

b *P* value for difference in means between teacher types calculated by linear and logistic mixed effects models accounting for clustering by teacher and school; *P* < .05 indicates significance.

c Lesson observation began when 50% of students had arrived at the PE area.

Compared with nonspecialist schools, students at specialist schools spent more time in skill development (21% vs 3%, *P* < .001) and less time in game play (18% vs 38%, *P* = .003). This difference persisted when comparing classroom teachers at specialist schools with classroom teachers at nonspecialist schools (Table 3).

### Middle and high schools

According to master schedules, all middle and high schools met the mandate of 200 minutes of PE per week; seventh graders had a mean 237 minutes and ninth graders had a mean 234 minutes of scheduled PE per week. (Because of block scheduling at middle and high schools, teacher schedules conformed to master schedules.) Only 3% of PE classes in middle schools were no-shows, and high schools had no no-shows. Observed lesson length in both middle and high schools was approximately 10 minutes shorter than scheduled lesson length (*P* < .001) (Table 2).

Seventh and ninth graders spent 51% and 56% of observed lesson time in moderate-to-vigorous physical activity, respectively (Table 2), which is equal to an average of 98 minutes per week from PE for seventh graders and 114 minutes for ninth graders.

Middle school PE lessons were largely spent in management tasks such as giving directions and behavior management (31% of lesson time) and free play or other activities (27% of lesson time). High school lessons were spent primarily in fitness activities such as running, stretching, and calisthenics (33% of lesson time) and free play or other (28% of lesson time)

Teacher characteristics (sex, years of PE teaching experience, self-reported enjoyment of teaching PE, training, and enjoyment for being physically active) did not predict minutes of PE per week or proportion of lesson time devoted to moderate-to-vigorous physical activity.

## Discussion

Although seventh- and ninth-grade students had PE scheduled according to California state mandates, schedules for fifth graders fell far short of the required minutes, corroborating previous research showing that PE is underscheduled at the elementary-school level based on school administrator report (6,7,15) and extending the evidence of noncompliance by demonstrating a further deficiency in PE minutes on the basis of direct observation. Among the 12 elementary schools that had a master PE schedule, teachers uniformly scheduled fewer minutes of PE than master schedules indicated. Given that many published reports of PE minutes rely on school principal or district-level administrators rather than teacher estimates (6,15,16), current reports likely overestimate the number of actual minutes of PE students receive. Furthermore, the observed 33% no-show proportion for PE in elementary schools suggests that published reports based on self-report could overestimate time in PE by as much as 50%.

During observed PE lesson time, students at all grade levels exceeded the recommended 50% of lesson time in moderate-to-vigorous physical activity. Although this high proportion has been previously demonstrated (17,18), most research shows that students spend less than 50% of class time in moderate-to-vigorous physical activity (19–22). Several factors could help explain our higher observed levels: grades observed (other studies observed younger students or multiple grades within a school-level) (19,20), number of lessons observed (we observed up to 3 lessons per teacher, as opposed to only 1) (19), sex (we observed both boys and girls) (22), and geographic variation (ours was a temperate climate) (19,20,22).

Because PE occurred infrequently for fifth graders, it did not substantially contribute to the recommended 60 minutes of daily moderate-to-vigorous physical activity (23); students received an average of only 36 minutes per week from PE classes. Although much PE research has focused on increasing student moderate-to-vigorous physical activity levels when class occurs (20,21), to our knowledge, interventions to increase PE policy implementation and adherence to time requirements have not been developed and rigorously tested. Further research is needed to identify viable methods for increasing mandate compliance.

In our study we did not find differences in moderate-to-vigorous physical activity related to the presence of a PE specialist. This finding, suggesting that nonspecialists were as successful as PE specialists in engaging students in moderate-to-vigorous physical activity, is encouraging. An observational study involving third-grade PE lessons also found no significant difference in mean moderate-to-vigorous physical activity between PE specialists and nonspecialists (19). However, the multicenter randomized Child and Adolescent Trial of Cardiovascular Health (24) demonstrated greater levels of moderate-to-vigorous physical activity in specialist-led PE. That study also demonstrated that exposure to PE specialists was associated with more time spent developing motor skills, as we found in our study. This is an important finding given that fundamental skills taught in PE have been shown to predict higher levels of participation in organized physical activity during adolescence (25). Notably, even classroom teachers in specialist schools spent more time developing students’ motor skills than teachers in nonspecialist schools, suggesting a positive effect of classroom teachers’ exposure to training by PE specialists. If budget constraints continue to limit schools’ abilities to hire full-time PE specialists, further work should identify best practices for sharing PE specialists’ time across schools.

At the middle and high school levels, students spent a considerable proportion of PE lesson time in free play and virtually no time in skills development. Although free play can provide an excellent opportunity for moderate-to-vigorous physical activity (26), it relies on students’ self-motivation to engage in activity. Students who enjoy being active may get more moderate-to-vigorous physical activity during free play than students who do not like to be active, students who are overweight, or students who have few physical skills (27). An increased focus on skills development or structured noncompetitive game play could increase PE’s reach.

Consistent with other studies, time was lost at the beginning and end of most scheduled PE lessons for transitioning from classroom to playground or changing clothes (in middle and high school) (20). Researchers have examined innovative ways to decrease changing time between classes and maximize scheduled PE time to increase the physical activity that occurs during PE, including using instant activities that take place as soon as students enter class and using music during changing time to encourage quicker transitions (28,29).

Although previous research has shown that lower-resource schools have less or poorer quality PE (30), neither teacher- nor school-level demographic information was associated with PE minutes or student moderate-to-vigorous physical activity levels in this study. However, all schools had diverse student bodies and a high proportion of students eligible for free or reduced-price meals.

One limitation of this study is its restriction to a single school district and a relatively small sample, which may limit the generalizability of the results. However, the study district’s size, demographic diversity, and urban location make it comparable to many districts across the state and country, and we expect that our finding that reported minutes of PE are greater than observed minutes would be readily reproduced in other districts. Although we did not use objective measures of moderate-to-vigorous physical activity, such as accelerometers, we did use a systematic observation system that has been widely used in PE research, allowing for the comparison of effect sizes across studies. Finally, the cross-sectional nature of this study does not allow us to draw causal relationships.

In this study, elementary schools did not meet California state PE requirements, and teachers did not regularly adhere to PE schedules. Noncompliance was much greater when PE time was measured objectively than when assessed by self-report (master schedules), suggesting that more accurate measures of PE reporting are necessary. New methods to assess PE policy compliance, such as having district administrators systematically collect PE data, need to be developed and validated. Middle and high schools did meet the PE mandate. Block schedules, with clearly laid out class times and bell schedules, may help ensure that PE occurs with regularity. It is promising that at the elementary-school level classroom teachers can successfully engage students in moderate-to-vigorous physical activity during PE. However, even if students are able to achieve a high proportion of PE lesson time in moderate-to-vigorous physical activity, PE may do little to contribute to students’ overall health if adequate PE minutes are not scheduled and teachers do not adhere to schedules. Further research is needed to determine best practices for increasing compliance with PE policy so that elementary students receive adequate PE.

## References

[1] US Department of Health and Human Services. Physical activity and health: a report of the Surgeon General. Atlanta (GA): US Department of Health and Human Services, Centers for Disease Control and Prevention, National Center for Chronic Disease Prevention and Health Promotion; 1996.

[2] Eaton DK, Kann L, Kinchen S, Shanklin S, Ross J, Hawkins J, et al. Youth Risk Behavior Surveillance—United States, 2009. MMWR Surveill Summ 2010;59(5):1–142. 20520591

[3] Madsen KA , Gosliner W , Woodward-Lopez G , Crawford P . Physical activity opportunities associated with fitness and weight status among adolescents in low-income communities. Arch Pediatr Adolesc Med 2009;163(11):1014–21. 10.1001/archpediatrics.2009.181 19884592 PMC3004432

[4] Trudeau F , Shephard RJ . Contribution of school programmes to physical activity levels and attitudes in children and adults. Sports Med 2005;35(2):89–105. 10.2165/00007256-200535020-00001 15707375

[5] California State Board of Education Policy No. 99–03. Education Code Section 51210. June 1999. http://www.cde.ca.gov/be/ms/po/policy99-03-june1999.asp. Accessed December 15, 2012.

[6] Sanchez-Vaznaugh EV , Sánchez BN , Rosas LG , Baek J , Egerter S . Physical education policy compliance and children’s physical fitness. Am J Prev Med 2012;42(5):452–9. 10.1016/j.amepre.2012.01.008 22516484

[7] California Department of Education. Compliance monitoring. http://www.cde.ca.gov/ta/cr/. Accessed December 7, 2012.

[8] US Department of Health and Human Services, Centers for Disease Control and Prevention. Strategies to improve the quality of physical education. National Center for Chronic Disease Prevention and Health Promotion, Division of Adolescent and School Health 2010:1-3. http://www.cdc.gov/healthyyouth/physicalactivity/pdf/quality_pe.pdf. Accessed December 15, 2012.

[9] McKenzie TL , Sallis JF , Faucette N , Roby JJ , Kolody B . Effects of a curriculum and inservice program on the quantity and quality of elementary physical education classes. Res Q Exerc Sport 1993;64(2):178–87. 10.1080/02701367.1993.10608795 8341841

[10] Pate RR , Davis MG , Robinson TN , Stone EJ , McKenzie TL , Young JC . Promoting physical activity in children and youth: a leadership role for schools: a scientific statement from the American Heart Association Council on Nutrition, Physical Activity, and Metabolism (Physical Activity Committee) in collaboration with the Councils on Cardiovascular Disease in the Young and Cardiovascular Nursing. Circulation 2006;114(11):1214–24. 10.1161/CIRCULATIONAHA.106.177052 16908770

[11] California Department of Education. Dataquest state education data reporting. http://data1.cde.ca.gov/dataquest/. Accessed March 1, 2012.

[12] McKenzie TL , Sallis JF , Nader RR . SOFIT: system for observing fitness instruction time. J Teach Phys Educ 1991;11:195–205.

[13] Scruggs PW , Beveridge SK , Eisenman PA , Watson DL , Shultz BB , Ransdell LB . Quantifying physical activity via pedometry in elementary physical education. Med Sci Sports Exerc 2003;35(6):1065–71. 10.1249/01.MSS.0000069748.02525.B2 12783057

[14] Lounsbery MAF , McKenzie TL , Morrow JR Jr , Holt KA , Budnar RG . School physical activity policy assessment. J Phys Act Health 2013;10(4):496–503. 22975809 10.1123/jpah.10.4.496

[15] Centers for Disease Control and Prevention. School Health Policies and Programs Study 2006, State-level school health policies and practices: a state-by state summary from the School Health Policies and Programs Study 2006. http://www.cdc.gov/healthyyouth/shpps/2006/summaries/pdf/State_Level_Summaries_SHPPS2006.pdf. Accessed December 18, 2012.

[16] Slater SJ , Nicholson L , Chriqui J , Turner L , Chaloupka F . The impact of state laws and district policies on physical education and recess practices in a nationally representative sample of US public elementary schools. Arch Pediatr Adolesc Med 2012;166(4):311-6. 22147763 10.1001/archpediatrics.2011.1133 PMC3523123

[17] Chow BC , McKenzie TL , Louie L . Children’s physical activity and environmental influences during elementary school physical education. J Teach Phys Educ 2008;27(1):38–50.

[18] McKenzie TL , Nader PR , Strikemiller PK , Yang M , Stone EJ , Perry CL , School physical education: effect of the Child and Adolescent Trial for Cardiovascular Health. Prev Med 1996;25(4):223–41. 10.1006/pmed.1996.0074 8818066

[19] Nader PR . Frequency and intensity of activity of third-grade children in physical education. Arch Pediatr Adolesc Med 2003;157(2):185–90. 10.1001/archpedi.157.2.185 12580690

[20] McKenzie TL , Sallis JF , Prochaska JJ , Conway TL , Marshall SJ , Rosengard P . Evaluation of a two-year middle-school physical education intervention: M-SPAN. Med Sci Sports Exerc 2004;36(8):1382–8. 10.1249/01.MSS.0000135792.20358.4D 15292747

[21] Stone EJ , McKenzie TL , Welk GJ , Booth ML . Effects of physical activity interventions in youth: review and synthesis. Am J Prev Med 1998;15(4):298–315. 10.1016/S0749-3797(98)00082-8 9838974

[22] McKenzie TL , Catellier DJ , Conway T , Lytle LA , Grieser M , Webber LA , Girls’ activity levels and lesson contexts in middle school PE: TAAG baseline. Med Sci Sports Exerc 2006;38(7):1229–35. 10.1249/01.mss.0000227307.34149.f3 16826019 PMC2431981

[23] US Department of Health and Human Services. Physical Activity Guidelines Advisory Committee Report. Washington (DC): US Department of Health and Human Services; 2008.

[24] McKenzie TL , Stone EJ , Feldman HA , Epping JN , Yang M , Strikmiller PK , Effects of the CATCH physical education intervention: teacher type and lesson location. Am J Prev Med 2001;21(2):101–9. 10.1016/S0749-3797(01)00335-X 11457629

[25] Okely AD , Booth ML , Patterson JW . Relationship of physical activity to fundamental movement skills among adolescents. Med Sci Sports Exerc 2001;33(11):1899–904. 10.1097/00005768-200111000-00015 11689741

[26] McKenzie TL , Feldman H , Woods SE , Romero KA , Dahlstrom V , Stone EJ , Children’s activity levels and lesson context during third-grade physical education. Res Q Exerc Sport 1995;66(3):184–93. 10.1080/02701367.1995.10608832 7481079

[27] Sallis JF , Prochaska JJ , Taylor WC . A review of correlates of physical activity of children and adolescents. Med Sci Sports Exerc 2000;32(5):963–75. 10.1097/00005768-200005000-00014 10795788

[28] Jago R , McMurray RG , Bassin S , Pyle L , Bruecker S , Jakicic JM , Modifying middle school physical education: Piloting strategies to increase physical activity. Pediatr Exerc Sci 2009;21(2):171–85. 19556623 10.1123/pes.21.2.171 PMC2705879

[29] McMurray RG , Bassin S , Jago R , Bruecker S , Moe EL , Murray T , Rationale, design and methods of the HEALTHY study physical education intervention component. Int J Obes (Lond) 2009;33 Suppl 4:S37–43. 10.1038/ijo.2009.115 19623187 PMC2747738

[30] UCLA Center to Eliminate Health Disparities and Samuels & Associates. Failing fitness: physical activity and physical education in schools. Los Angeles (CA): The California Endowment; 2007.

